# Enhancement of Xanthophyll Synthesis in *Porphyra/Pyropia* Species (Rhodophyta, Bangiales) by Controlled Abiotic Factors: A Systematic Review and Meta-Analysis

**DOI:** 10.3390/md19040221

**Published:** 2021-04-15

**Authors:** Florentina Piña, Loretto Contreras-Porcia

**Affiliations:** 1 Departamento de Ecología y Biodiversidad, Facultad de Ciencias de la Vida, Universidad Andres Bello, Santiago 8370251, Chile; florentinapina1996@gmail.com; 2 Centro de Investigación Marina Quintay (CIMARQ), Facultad de Ciencias de la Vida, Universidad Andres Bello, Quintay 2531015, Chile; 3 Center of Applied Ecology and Sustainability (CAPES), Santiago 8331150, Chile; 4 Instituto Milenio en Socio-Ecología Costera (SECOS), Santiago 8370251, Chile

**Keywords:** abiotic factors, carotenoids, lutein, *Porphyra*, *Pyropia*, xanthophylls, zeaxanthin

## Abstract

Red alga species belonging to the *Porphyra* and *Pyropia* genera (commonly known as Nori), which are widely consumed and commercialized due to their high nutritional value. These species have a carotenoid profile dominated by xanthophylls, mostly lutein and zeaxanthin, which have relevant benefits for human health. The effects of different abiotic factors on xanthophyll synthesis in these species have been scarcely studied, despite their health benefits. The objectives of this study were (i) to identify the abiotic factors that enhance the synthesis of xanthophylls in *Porphyra*/*Pyropia* species by conducting a systematic review and meta-analysis of the xanthophyll content found in the literature, and (ii) to recommend a culture method that would allow a significant accumulation of these compounds in the biomass of these species. The results show that salinity significantly affected the content of total carotenoids and led to higher values under hypersaline conditions (70,247.91 µg/g dm at 55 psu). For lutein and zeaxanthin, the wavelength treatment caused significant differences between the basal and maximum content (4.16–23.47 µg/g dm). Additionally, in *Pyropia* spp., the total carotenoids were considerably higher than in *Porphyra* spp.; however, the lutein and zeaxanthin contents were lower. We discuss the specific conditions for each treatment and the relation to the ecological distribution of these species.

## 1. Introduction

Carotenoids are lipophilic isoprenoid compounds with a wide range of properties and natural pigments in the yellow to red range, and they are synthesized by all photosynthetic organisms, including bacteria and fungi [1]. Typically, they contain 40 carbon atoms (C40) and are formed by eight C5 isoprenoid units. Based on their chemical composition, they can be divided into carotenes and xanthophylls.

The non-polar carotenoid molecules that contain only carbon and hydrogen atoms are called carotenes (e.g., α-carotene, β-carotene and lycopene), while the polar carotenoids are called xanthophylls, and they contain at least one oxygen atom (e.g., lutein and zeaxanthin) [2]. The consumption of dietary carotenoids, or foods rich in these pigments, are considered to be beneficial in the prevention of a variety of major diseases, including eye diseases, certain cancers, and neurodegenerative diseases [3,4,5].

Due to their antioxidant activity, xanthophylls such as lutein and zeaxanthin are associated with positive effects for human eye health [6]. Several reports suggested that the consumption of foods rich in these compounds may slow or even stop the progression of age-related macular degeneration (AMD) and cataracts [7]. In addition, recent clinical studies suggested that the daily consumption of lutein may slow the progression of neurodegenerative diseases such as Parkinson’s and Huntington’s disease [5]. On the other hand, due to their antioxidant capacity, lutein and zeaxanthin have also been associated with positive effects on cardiovascular health, certain types of cancer related with obesity, lung cancer, and breast cancer [8,9,10].

There is an increasing interest in the discovery of health-promoting substances of marine origin, particularly from marine seaweeds residing in the littoral zone, which are now considered primary resources of the ocean in terms of economic and ecological significance [11]. The red seaweed *Porphyra* and closely related genera *Pyropia* (known collectively by the Japanese name Nori or Laver) are important marine crops that were worth US $2.66 billion in 2019 with a production of 2,984,123 tons [12]. This significant economic interest is mainly due to the high nutritional and functional values, including important contents of vitamins, dietary fiber, protein, polyunsaturated fatty acids, mycosporine-like amino acids, and carotenoids, among others [13,14].

Carotenoids play a key role in photosynthetic organisms, as they allow a balance between absorbing enough light for photosynthetic processes, while avoiding photo-oxidative damage in the photosynthetic apparatus caused by excess light [15]. To achieve this balance, carotenoids such as xanthophylls can: (i) stabilize chlorophyll in the excited state, preventing the formation of reactive oxygen species (ROS); (ii) directly eliminate ROS (such as oxygen singlets); and (iii) dissipate excess energy via non-photochemical quenching (NPQ) mediated by zeaxanthin [16,17,18].

**Figure 1:**
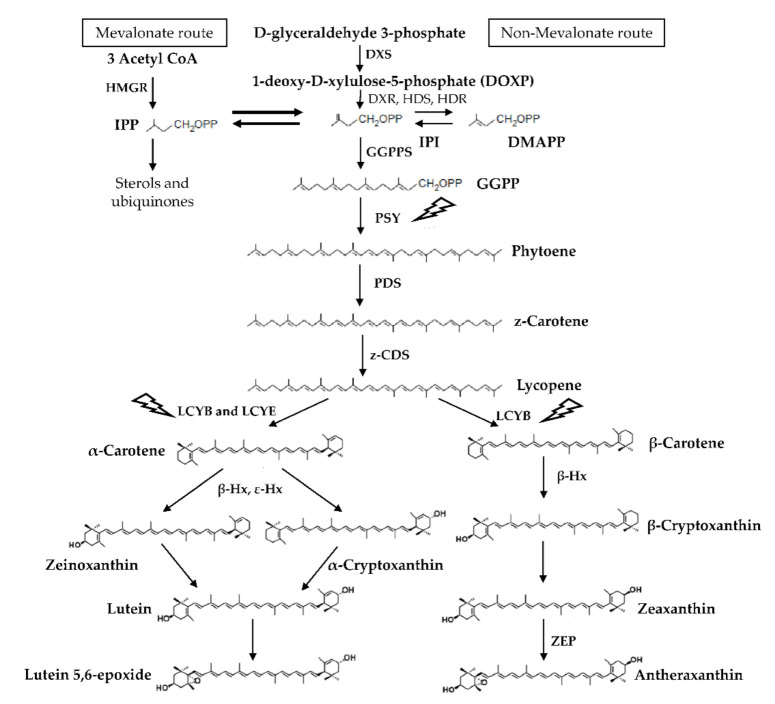
Schematic representation of xanthophyll synthesis in red algae. DOXP-synthase (DXS), DOXP reductoisomerase (DXR), isopentenyl pyrophosphate (IPP), dimethylallyl pyrophosphate (DMAPP), geranylgeranyl pyrophosphate (GGPP), GGPP synthase (GGPPS), phytoene synthase (PSY), phytoene desaturase (PDS), z-carotene desaturase (z-CDS), lycopene β-cyclase (LCYB), lycopene ε-cyclase (LCYE), and zeaxanthin epoxidase (ZEP). The lightning symbol indicates that light has an effect over the carotenogenic gene expression of the enzyme. The upstream lycopene synthesis pathway is as described for plants and algae, modified from [20]. Downstream of lycopene corresponds to the synthesis pathways particularly of *Pyropia yezoensis*, although enzymes unique to Bangiales have not yet been described; modified from [21].

In plants and algae, carotenoids are synthesized and accumulated in the thylakoid membranes, specifically, in the light harvesting complex [19]. The biosynthesis of xanthophylls begins with the synthesis of isopentenyl pyrophosphate (IPP), through two alternative routes: (i) the mevalonate acetate route and (ii) the non-mevalonate route, where xanthophylls are obtained as products (Figure 1). The main enzymes involved in this process are isopentenyl pyrophosphate synthase (IPI), geranylgeranyl pyrophosphate synthase (GGPPS), phytoene synthase (PSY), phytoene desaturase (PDS), lycopene-β-cyclase (LCYB), lycopene-ε-cyclase (LCYE), β- hydroxylase (β-Hx), and ε- hydroxylase (ε-Hx) [20].

Red algae contain several different carotenoids, including β-carotene, zeaxanthin, antheraxanthin, and lutein, with interspecific variation in their composition [22]. As carotenoids have specific roles as antioxidants in photoprotection, the different carotenoid profiles of red alga species are related to their ability to cope with oxidative and photo inhibitory stress, which determine their distribution in the intertidal zone [23,24].

Members of the class Bangiophyceae, such as the *Porphyra/Pyropia* species, have a carotenoid profile composed mainly of lutein, representing more than 50% of its total, as well as α-carotene, β-carotene, and zeaxanthin [25]. Therefore, these species have potential for the development of functional ingredients as a highly valuable food with respect to human health in association with the content of lutein and other xanthophylls.

The regulation of the biosynthesis of these compounds has been well studied in plants and cyanobacteria, and most of these studies determined that light plays a key role in this process, affecting the induction of the expression of carotenoid genes and the activity of key enzymes, such as PSY and PDS [26,27,28,29]. In algae, in addition to light, other abiotic factors, like temperature, salinity, and nitrogen deprivation were found to generate an accumulation of carotenoids in *Haematococcus pluvialis* and *Chlamydomonas reinhardtii* [30,31].

However, the effects of different abiotic factors on xanthophyll synthesis in commercially important seaweeds, such as *Porphyra/Pyropia* species, have been scarcely studied, despite the high consumption around the world of these food resources, the significant nutritional value of these genera, and the health benefits of these compounds. In this context, the aims of the present work were (i) to identify the abiotic factors that enhance the synthesis of xanthophylls in species of the *Porphyra* and *Pyropia* genera by conducting a meta-analysis of the xanthophyll content found in literature, and (ii) to recommend the standardization of a culture method that allows a significant accumulation of these compounds in a commercialized biomass of *Porphyra* and *Pyropia* species.

## 2. Results

### 2.1. Systematic Review and Data Extraction

Through the initial search, a total of 6901 articles were identified. After deleting duplicates, 3819 articles remained and were screened by title and abstract to remove irrelevant articles. The 117 articles remaining from the title and abstract screening were assessed for eligibility by a full-text revision based on the exclusion criteria (Table 1).

**Figure 2:**
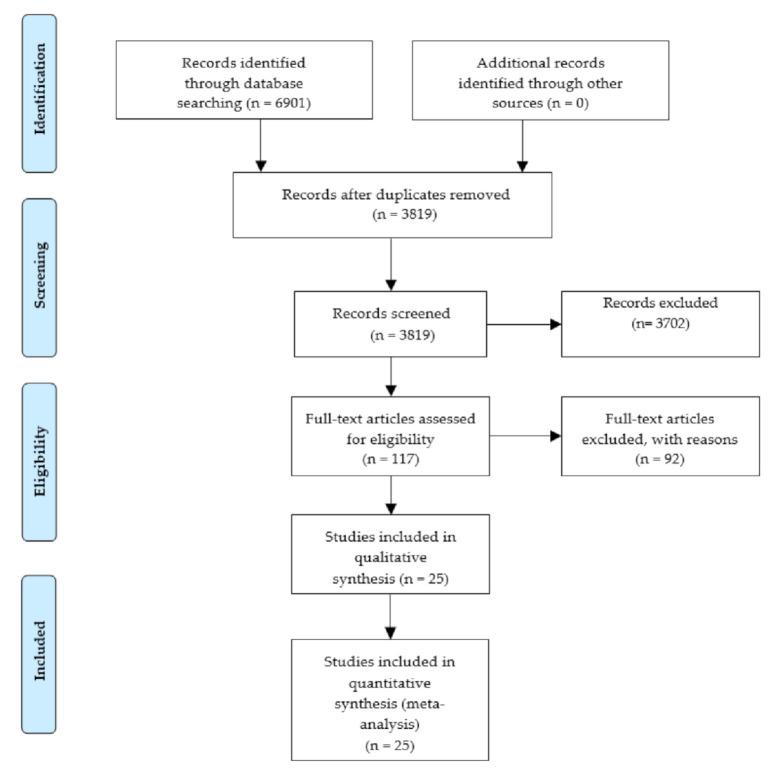
Flow of information through the different phases of the systematic review.

A total of 25 articles were included for the data extraction and meta-analysis, 9 of which were for the genus *Porphyra,* and 16 were for the genus *Pyropia* (Figure 2). In addition, we identified that 18 of the 25 included articles used algae from natural grassland harvesting, and the remainder sourced algae from controlled aquaculture.

The publication years of the articles ranged between 1999–2020. Four species of *Porphyra* were identified with the most frequent being *Porphyra haitanensis* (*n* = 4). For the genus *Pyropia*, five species were identified with *Pyropia yezoensis* as the most frequent (*n* = 6) (Table 2).

**Figure 3:**
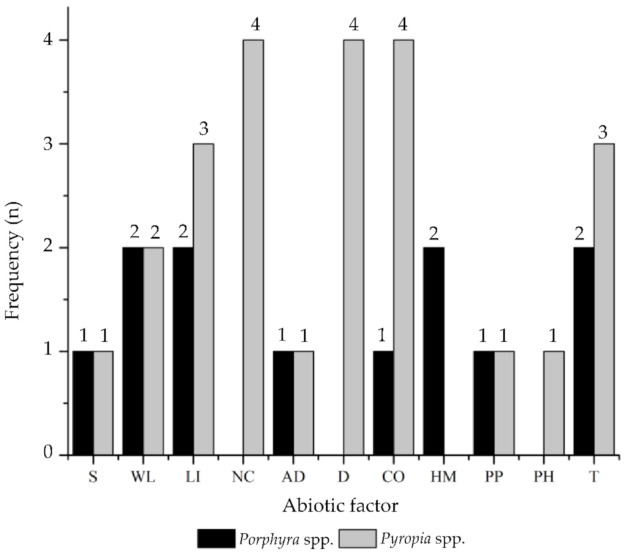
Frequency of the number of articles according to the abiotic factors identified for *Porphyra* and *Pyropia* species. Abiotic factors: salinity (S), wavelength (WL), light intensity (LI), nutrient composition (NC), algal density (AD), desiccation (D), CO_2_ (CO), heavy metals (HM), photoperiod (PP), pH (PH), and temperature (T).

Through data extraction, a total of 11 abiotic factors and 15 treatments were identified, as reported in the methodologies of each article (Table 3). According to the distribution of the abiotic factors, there was a higher frequency for the *Pyropia* species, with the most recurrent factors being the nutrient composition (NC), desiccation (D), and CO_2_ (CO) (*n* = 4). For the *Porphyra* species, the factors of wavelength (WL), light intensity (LI), heavy metals (HM), and temperature (T) had the highest frequency (*n* = 2). Overall, for both genera, the light intensity, CO_2_, and temperature were the most representative abiotic factors (Figure 3).

Regarding the ‘measured compound’ variable, only three items were identified, with the total carotenoids being the most frequent (*n* = 21), followed by lutein (*n* = 5) and zeaxanthin (*n* = 3). Spectrophotometry was the method used for the quantified compounds in 20 of the 25 articles included. In the remaining articles, either high performance liquid chromatography coupled with a UV detector (HPLC-UV) or ultra HPLC (UHPLC) were used, grouped under the item LC-UV.

### 2.2. Meta-Analysis

A total of 421 data points were extracted and included in the meta-analysis for the variable ‘experimental content’ (including the minimum, basal, and maximum values). The carotenoid profile for the *Porphyra* and *Pyropia* species was mainly characterized for the compound ‘total carotenoids’, representing 98.8% of the total, followed by ‘lutein’ (1.01%) and ‘zeaxanthin’ (0.01%).

**Figure 4:**
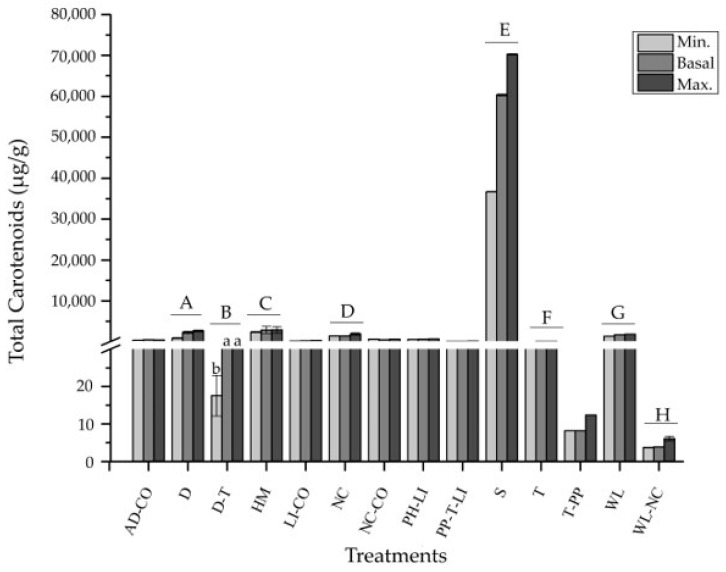
The total carotenoids (µg/g dm) minimum, basal, and maximum experimental content for *Porphyra*/*Pyropia*. The mean ± standard error (SE) of the extracted data is presented (*n* ≥ 3). Different capital letters above the bars indicate significant differences between treatments, and lower-case letters indicate significant differences between contents for the same treatment, with a *p* < 0.05 according to the generalized linear model (GLM). Treatments: algal density and CO_2_ (AD-CO); desiccation (D); desiccation and temperature (D-T); heavy metals (HM); light intensity and CO_2_ (LI-CO); nutrient composition (NC); nutrient composition and CO_2_ (NC-CO); pH and light intensity (PH-LI); photoperiod, temperature, and light intensity (PP-T-LI); salinity (S); temperature (T); temperature and photoperiod (T-PP); wavelength (WL); and wavelength and nutrient composition (WL-NC).

The total carotenoid experimental content was significantly affected by the treatments of desiccation (D), desiccation and temperature (D-T), heavy metals (HM), nutrient composition (NC), salinity (S), temperature (T), wavelength (WL), and wavelength and nutrient composition (WL-NC) (*p* < 0.05; according to the generalized linear model (GLM)) (Figure 4). The highest total carotenoid content values were recorded under the S treatment, registering 70,247.91 µg/g dm.

While all the treatments generated a neutral or positive effect on the total carotenoid content, no significant differences were found between the basal and maximum contents (Figure 4).

**Figure 5:**
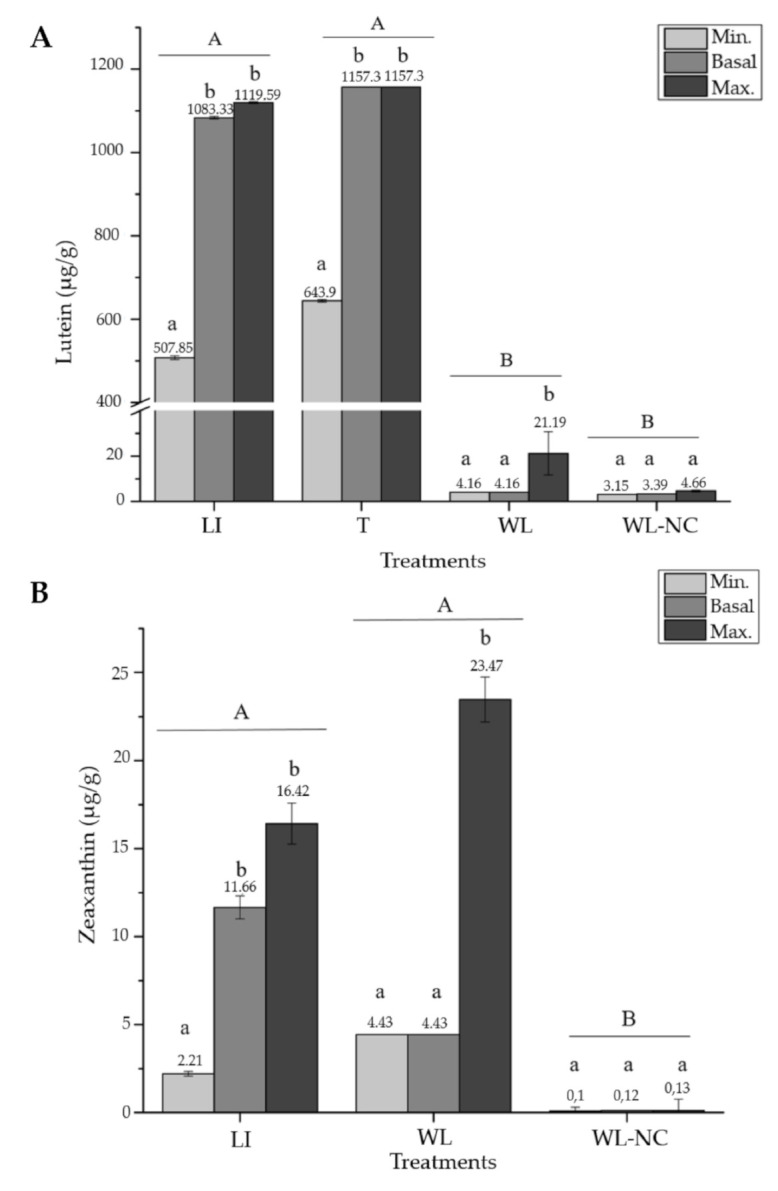
The minimum, basal, and maximum experimental content (µg/g dm) for *Porphyra/Pyropia* species of (**A**) lutein and (**B**) zeaxanthin. The mean ± SE of the extracted data is presented (*n* ≥ 3). Different capital letters above the bars indicate significant differences between treatments, and lower-case letters indicate significant differences between contents for the same treatment, with a *p* < 0.05 according to the GLM. Treatments: light intensity (LI), temperature (T), wavelength (WL), and wavelength and nutrient composition (WL-NC).

For the xanthophyll lutein, experimental content results were only reported under the light intensity (LI), temperature (T), wavelength (WL), and wavelength and nutrient composition (WL-NC) treatments (Figure 5A). Significant differences were found between the basal and maximum content only under the WL treatment (*p =* 0.0887). The highest lutein values were recorded under LI and T treatments at 1119.58 µg/g dm and 1157.3 µg/g dm, respectively (Figure 5A).

In the case of zeaxanthin, the LI and WL treatments showed significant differences with respect to the WL-NC treatment and the experimental content (*p* < 0.05; according to the GLM). Although the maximum zeaxanthin content was higher than the basal content in all treatments, this increase was only significant under the WL treatment (Figure 5B, *p =* 0.0036). The maximum value reached in this treatment was 23.47 µg/g dm.

**Figure 6:**
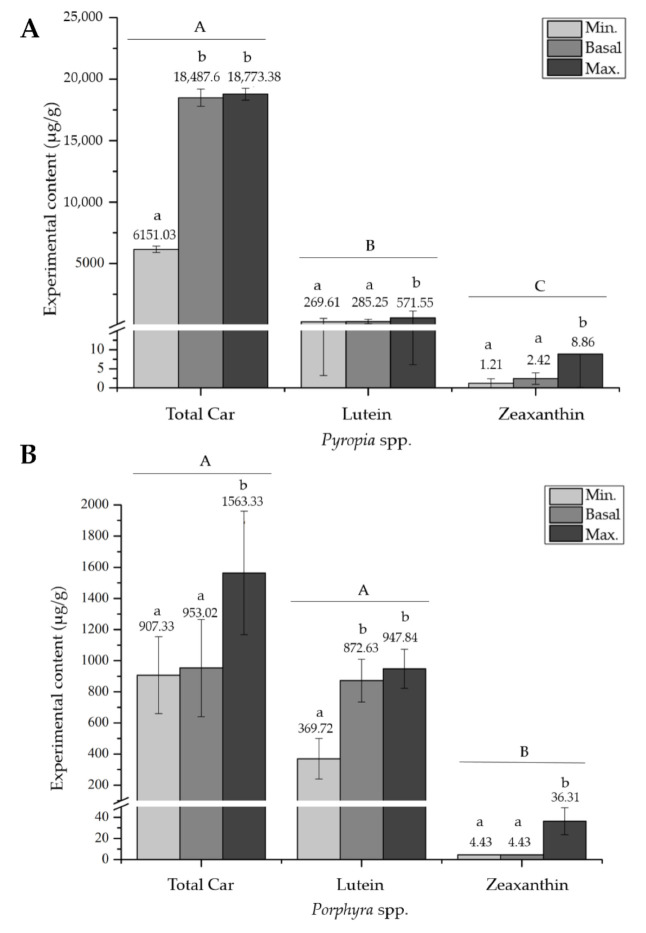
The minimum, basal, and maximum experimental content (µg/g dm) of the total carotenoids, lutein, and zeaxanthin for (**A**) *Pyropia* species and (**B**) *Porphyra* species. The mean ± SE of the extracted data is presented (*n* ≥ 3). Different capital letters above the bars indicate significant differences between treatments and lower-case letters indicate significant differences between contents for the same treatment, with a *p* < 0.05 according to the GLM.

Significant differences (*p =* 0.0025) were found between *Porphyra* and *Pyropia* species. For the species of the genus *Pyropia*, the content of the total carotenoids, lutein, and zeaxanthin were significantly different between them (Figure 6). Additionally, significant differences were evidenced between the minimum and maximum content for the three compounds, and between the basal and maximum content for lutein and zeaxanthin (*p* < 0.05, according to the GLM) (Figure 6A). For the species of the *Porphyra* genus, significant differences were only evident between the total carotenoid and lutein content compared to zeaxanthin (*p =* 0.0153). On the other hand, only for the total carotenoids and zeaxanthin, the maximum content was significantly higher than the basal value (Figure 6B).

Overall, the total carotenoid content was 31.23 times higher in *Pyropia* spp. than in *Porphyra* spp. However, the lutein content was 1.34 times higher in *Porphyra* spp., as well as the zeaxanthin content, which was 4.1 times higher.

### 2.3. Suggested Culture Method

The findings of this study suggest that salinity (S) and wavelength (WL) are important treatments to consider for the accumulation of xanthophylls, such as lutein and zeaxanthin, as well as the total carotenoids in both genera, since these abiotic factors had a significant positive effect on the content of these compounds.

**Figure 7:**
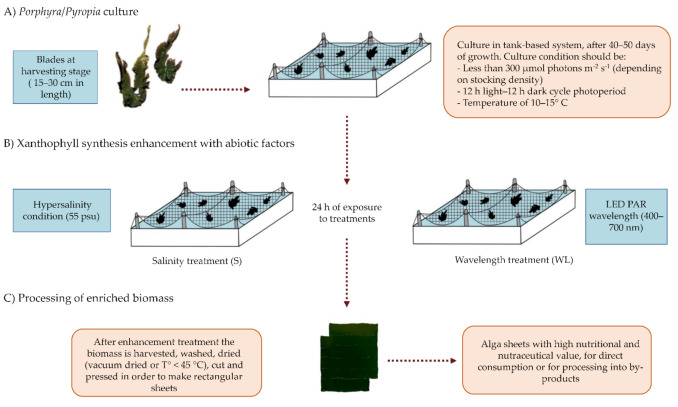
Scheme of the suggested culture method, based on three stages: (**A**) initial *Porphyra/Pyropia* species culture, (**B**) enhancement of compounds with abiotic factors (salinity or wavelength), and (**C**) processing of the enriched biomass.

According to the full-text revision of each article included in this systematic revision, to obtain the maximum experimental content of the total carotenoids, species of *Pyropia/Porphyra* should be cultivated in hypersaline conditions of 55 psu for a maximum period of 24 h (Figure 7A). Other culture conditions considered should include 15 °C and 100 µmol photons m^−2^ s^−1^ of photosynthetic active radiation (PAR) with a 12 h light/12 h dark cycle.

However, due to the significantly higher content of the total carotenoids in *Pyropia* spp. compared to *Porphyra* spp. (Figure 6), we recommend the use of species of this genus to obtain even higher content values of these compounds. To maximize the content of xanthophylls, the algae should be exposed to PAR wavelengths (400–700 nm) at 60 µmol photons m^−2^ s^−1^ and a 12 h light/12 h dark cycle (Figure 7B).

As a result of these treatments, an enriched algal biomass will be obtained with high nutritional value, suitable for drying and direct consumption, or for processing into by-products (Figure 7). Additionally, as shown in Figure 4, the drying and temperature treatment (D-T) caused a significant decrease in the total carotenoids when the drying technique was other than vacuum and the temperature was higher than 45 °C. Therefore, to avoid impairing the effect of abiotic factors on the stimulation of these compounds, these parameters should be considered when processing the enriched biomass (Figure 7C).

## 3. Discussion

The results obtained showed that the carotenoid profile of the *Porphyra*/*Pyropia* species was mainly dominated by the total carotenoid compounds, followed by lutein and zeaxanthin. Although the total carotenoids include the content of all carotenoid compounds present in algae (carotenes and xanthophylls), it is highly probable that up to 90% of this total content corresponds to the xanthophyll compound lutein.

In Rhodophyta, three main groups of carotenoid profiles have been identified [22], and, for species containing lutein as the main compound, they have been referred to as the LUT-group. In species where zeaxanthin is the predominant carotenoid, they are referred to as group-ZEA, and they are referred to as the ATX-group when antheraxanthin dominates the carotenoid profile [23]. As reported in the literature, the carotenoid profile in Bangiophyceae is composed of species of the ZEA-group and the LUT-group, where analysis of several species of this family (including *Porphyra perforata* and *Porphyra yezoensis*), showed that, in all species, lutein was the most abundant carotenoid, constituting between 50–90% of the total composition [25].

Latorre et al. (2019) reported similar results in the genus *Pyropia*, particularly in *Pyropia orbicularis,* where the compound lutein characterizes the bioactive fraction of this species and was suggested as the main compound for its antioxidant activity [57]. Therefore, the carotenoid profile reported in this systematic review and metric analysis agrees with the literature, and we suggest that the predominant percentage of the compounds identified in this study (as the total carotenoid) was lutein.

Species of the *Porphyra* and *Pyropia* genera inhabit highly dynamic environments (the intertidal zone), where abiotic factors play a key role in modulating their ecological distribution (e.g., the temperature, irradiance, and salinity) [58]. Consequently, these species have developed effective mechanisms to counteract the oxidative stress damage that these factors can trigger [59]. In this scenario, salinity is one of the abiotic factors that differs most with respect to time and space, as intertidal algae are exposed to extreme variations in salinity on time scales of seasons, days, or even hours [59].

For example, in intertidal pools, extreme salinity can occur at low tide due to evaporation. In this study, we determined that the total carotenoid content was considerably higher under the salinity treatment. According to Samanta et al. (2019), the carotenoid content in *Pyropia yezoensis* was higher under hypersalinity condition (55 psu) compared to the control (30 psu), indicating that this species possesses an adaptation to hypersalinity mediated by carotenoid compounds [48].

This response could be closely related to the hypersalinity-induced stress resulting from the natural tidal cycle to which the *Porphyra*/*Pyropia* species are exposed, where the increase of carotenoids would be part of the antioxidant defense strategy to eliminate reactive oxygen species (ROS) in the short term. This has been determined to be the case in *Dunaliella salina*, *Scenedesmus* sp., and the red macroalga *Acanthophora spicifera* [60].

As for the effect of the different treatments on the lutein and zeaxanthin contents, this study showed that only the WL treatment generated a significant increase compared to the control or basal content. Previous studies have demonstrated that the compound zeaxanthin has several photoprotective mechanisms mainly associated with the photosystem I (PSI), and the synthesis of this compound has multiple effects [61]. For instance, this compound induces energy dissipation when the organism is exposed to excess energy (high light intensity or wavelength), and it reduces the ROS produced by pigment–protein complexes [61,62]. In addition, increased lutein synthesis was also described as an efficient photoprotection strategy, as lutein has the ability to work as a direct energy quencher for excited chlorophylls [23].

Several studies have shown an increase in the synthesis of xanthophylls, such as lutein and zeaxanthin grown under PAR wavelengths when compared to ultraviolet A (UVAR, 365 nm) and ultraviolet B radiation (UVBR, 312 nm), which produced a decrease in the contents of these compounds for the *Porphyra*/*Pyropia* species. Pereira et al. (2020) showed that the content of total carotenoids was significantly higher under PAR light treatment compared to PAB (UVBR) in *Pyropia acanthophora* var. *brasiliensis* [44]. This study also showed that lutein was the main carotenoid, and, to favor its synthesis, the presence of NO_3_^−^ in the culture medium is required.

To produce this compound, the hydroxylation of α-carotene must occur, and certain nutrients present in the external environment are required, as reported in *Porphyra leucosticta* [63]. Similarly, Bouzon et al. (2012) found that the total carotenoid content significantly increased from 57.8 to 315 µg/g in *Porphyra acanthophora* var. *brasiliensis* when treated with PAR light. The authors demonstrated that this treatment induced the accumulation of these compounds, and they suggested that the response can be interpreted as a photoprotective acclimation mechanism favored by culture conditions including enriched medium and PAR light [38].

Regarding the present study, the overall carotenoid content (including xanthophylls) was considerably higher in *Pyropia* spp. than in *Porphyra* spp. This difference between genera, whose distribution often overlaps in the intertidal zone, could be explained by the differences between the micro-ecological niches of the species. Meynard et al. (2019) found that, in the intertidal zone, the species *Porphyra luchea* and *Pyropia variabilis* were generally found living in sympatry.

However, in certain microhabitats, such as rock walls, a single dominant species (e.g., *P. variabilis*) was observed [64]. This could be related to differences in the degree of tolerance or the physiological adaptations to abiotic factors between those species [65]. In this scenario, we suggest that *Pyropia variabilis* better tolerates abiotic factors compared with *Porphyra luchea*, since the former dominates microhabitats, such as rock walls, where the sun exposure is higher than in boulder areas surrounded by intertidal pools (in terms of the light intensity and wavelength); and this is likely related to the differences in their carotenoid profiles.

In conclusion, xanthophylls are the main carotenoids present in the *Porphyra*/*Pyropia* species included in this work, and, under stress conditions triggered by abiotic factors such as salinity and wavelength, the content of these compounds tends to increase compared to the basal content. Therefore, growing *Porphyra* spp. and/or *Pyropia* spp. under the conditions suggested in this study (Figure 7) favors the production of compounds with high biological activity, which is sought after for the food and nutraceutical industries [43]. Adding value to the cultivated biomass by generating a product with direct benefits for human health makes the option of culturing these resources for productive purposes more attractive.

## 4. Materials and Methods

### 4.1. Systematic Review

From July–October 2020, we searched studies related to the basal content and the effects of different abiotic factors on the content of xanthophylls in species belonging to the *Porphyra* and *Pyropia* genera, using the electronic databases Crossref, Google Scholar, Scopus, and Web of Science. The search strategy was based on keywords and the following algorithms: (i) *Porphyra* or *Pyropia* and xanthophyll and abiotic factor, (ii) *Porphyra* or *Pyropia* and xanthophyll or carotenogenesis and abiotic factor, (iii) *Porphyra* or *Pyropia* and lutein or zeaxanthin and abiotic factor, (iv) Laver or Nori and xanthophyll and abiotic factor, (v) Laver or Nori and carotenogenesis or xanthophyll. Additionally, the references of the articles found were manually checked for further relevant articles. The flow of information of the systematic review according to the PRISMA statement [66] is described in Figure 2.

The articles identified by the search criteria were selected according to language filters, selecting only articles in English, Spanish, or Portuguese, while a filter by year of publication was not included. Further selection consisted of the title and abstract revision, to exclude articles that did not contain any of the above-mentioned keywords in the title or abstract of the article. To assess the eligibility of the articles, a full-text revision was performed by one reviewer (F.P.) and a second reviewer (L.C.-P.) was consulted when necessary. Only those articles that met none of the exclusion criteria (Table 1) were included in the data analysis.

### 4.2. Data Extraction

Data were extracted and recorded from each article to be included in the meta-analysis regarding the following variables: the (i) year of article publication, (ii) genus, (iii) species, (iv) measured compound (‘measured compound’), (v) abiotic factor used as a control condition (‘control factor’), (vi) content of the measured compound in the control condition (‘control content’), (vii) abiotic factor used as an experimental condition (‘experimental factor’), (viii) content of the measured compound in the experimental condition (‘experimental content’), (ix) time of exposure, and (x) measuring method for the identified compound (‘measuring method’).

The extracted categorical variables were classified according to the information provided by each of the included articles (Table 3). Due to taxonomic updates for some of the species in these genera, a review of the status of the scientific names was conducted for each of the included species [67]. However, no adjustments were required as all of the included species were taxonomically accepted at the time of this review.

In the case of the variable ‘experimental factor’, classifications were used to group the identified abiotic factors. For example, the classification of “heavy metals” was used, which was composed of Cu, Cd, and Zn. For the variable ‘measuring method’, the classification “LC-UV” was composed of HPLC-UV (high performance liquid chromatography coupled with a UV detector) and UHPLC (ultra HPLC).

For the quantification of the number of articles per ‘experimental factor’ variable, we assigned presence and absence values according to the classification of the variable designated for each abiotic factor individually. To evaluate the effect of the diverse abiotic factors on the experimental content, the variable ‘treatment’ was assigned, including the abiotic factors as individual or mixed as described from each article (Table 3).

For continuous variables, such as ‘experimental content’, the minimum, basal (equivalent to the value of the ‘control content’), and maximum value (the largest basal value) were recorded, and all of them were standardized to µg/g of dry mass (dm), by means of the corresponding conversion applied to the raw data extracted for each included article.

### 4.3. Meta-Analysis

The statistical analyses involved the elaboration of histograms and multivariable analyses. The histograms were used in combination with the Kolmogorov–Smirnov test with the Lilliefors correction to determine the distribution of each variable of interest. Then, a generalized linear model (GLM) was used to demonstrate the significant differences between each of the identified treatments and the experimental content (minimum, basal, and maximum) for each measured compound. Subsequently, the existence of significant differences between the ranges of experimental content (minimum and maximum) of each compound according to the genus was evaluated, thus separating the data obtained for the species of *Porphyra* and *Pyropia*. All the analyses were carried out in the software RStudio Team (4.0.0) [68].

The effect size (ES) of each study could not be calculated, due to the lack of specification of the sample size (*n*) in most of the studies included in the meta-analysis, as, when measuring the content of certain compounds, the sample amount was expressed in grams (g, dry mass or fresh weight), and the weight of individual organisms varies between species. Consequently, the publication bias could not be assessed [69].

## Figures and Tables

**Table 1:** Exclusion criteria for the articles and the number of articles excluded.

Exclusion Criteria	Articles Excluded
1. Species different from *Porphyra* spp. or *Pyropia* spp. were used.	11
2. Carotenoid content was not reported.	50
3. The experimental factor evaluated was a biological factor.	1
4. The carotenoid content was evaluated at baseline, without the alteration of an experimental factor.	13
5. The methodology of the experimental factor or treatment was not descriptively explained.	1
6. The control condition was not reported.	3
7. The magnitude of the experimental factor was not specified.	1
8. Experimental factor or treatment was evaluated on the conchocelis, spores or reproductive structures.	6
9. Article was not available in electronic databases.	6

**Table 2:** List of the identified species. The compound measured in each species, experimental content range (µg/g dm) and measure method with the corresponding references.

Genus	Specie	Total Carotenoids	Lutein	Zeaxanthin	Measure Method	Ref.
*Porphyra*	*Po. haitanensis*	62.5–10,000	-	-	SP ^1^	[32,33,34,35]
	*Po.* sp.	-	430–1117	-	LC-UV ^2^	[36,37]
	*Po. acanthophora* var. *brasiliensis*	-	4.16–30.71	4.43–36.31	LC-UV	[38]
	*Po. umbilicalis*	+	-	-	SP	[39,40]
*Pyropia*	*Py. orbicularis*	2.2–170.84	-	-	SP	[41,42]
	*Py. acanthophora* var. *brasiliensis*	3.63–12.4	3.22–6.1	0.07–0.15	SP/LC-UV	[43,44]
	*Py. yezoensis*	225–2.2 × 10^4^	536–1137	2.36–17.58	SP/LC-UV	[45,46,47,48,49,50,51]
	*Py. spiralis*	500–1.5 × 10^4^	-	-	SP	[52]
	*Py. haitanensis*	280–800	-	-	SP	[53,54,55,56]

^1^ SP = Spectrophotometry. ^2^ LC-UV = Liquid chromatography coupled to ultraviolet detection.

**Table 3:** Classification of the categorical variables and the number of articles.

Variable	Classification of Variables	N° of Articles
Genus	*Porphyra*	9
	*Pyropia*	16
Measured compound	Total carotenoids	21
	Lutein	5
	Zeaxanthin	3
Measure method	LC-UV	5
	Spectrophotometry	20
Experimental factor	Salinity (S)	2
	Wavelength (WL)	4
	Light Intensity (LI)	5
	Nutrients Composition (NC)	4
	Algal/Culture Density (AD)	2
	Desiccation (D)	4
	CO_2_ (CO)	5
	Heavy Metals (HM)	2
	Photoperiod (PP)	2
	pH (PH)	1
	Temperature (T)	5
Treatments	S	2
	WL	3
	WL-NC	1
	LI	2
	LI-CO	1
	NC	1
	NC-CO	2
	AD-CO	2
	D	3
	D-T	1
	HM	2
	PP-T-LI	1
	PH-LI	1
	T	2
	T-PP	1

## Data Availability

Derived data supporting the findings of this study are available from the corresponding author L. Contreras-Porcia on request.
